# The contact system in liver injury

**DOI:** 10.1007/s00281-021-00876-7

**Published:** 2021-06-14

**Authors:** Chandini Rangaswamy, Reiner K. Mailer, Hanna Englert, Sandra Konrath, Thomas Renné

**Affiliations:** Institute of Clinical Chemistry and Laboratory Medicine (O26), University Medical Center Hamburg-Eppendorf, Martinistrasse 52, D-20246 Hamburg, Germany

**Keywords:** Contact system, Coagulation, Factor XII, Liver disease, Inflammation

## Abstract

Coagulation is controlled by a delicate balance of prothrombotic and antithrombotic mechanisms, to prevent both excessive blood loss from injured vessels and pathologic thrombosis. The liver plays a pivotal role in hemostasis through the synthesis of plasma coagulation factors and their inhibitors that, in addition to thrombosis and hemostasis, orchestrates an array of inflammatory responses. As a result, impaired liver function has been linked with both hypercoagulability and bleeding disorders due to a pathologic balance of pro- and anticoagulant plasma factors. At sites of vascular injury, thrombus propagation that finally may occlude the blood vessel depends on negatively charged biopolymers, such as polyphosphates and extracellular DNA, that provide a physiological surface for contact activation of coagulation factor XII (FXII). FXII initiates the contact system that drives both the intrinsic pathway of coagulation, and formation of the inflammatory mediator bradykinin by the kallikrein–kinin system. Moreover, FXII facilitates receptor-mediated signalling, thereby promoting mitogenic activities, angiogenesis, and neutrophil stimulation with implications for liver diseases. Here, we summarize current knowledge on the FXII-driven contact system in liver diseases and review therapeutic approaches to target its activities during impaired liver function.

## Introduction

Blood coagulation not only is a physiological process required to prevent blood loss following vessel injury, but also contributes to the formation of thrombi that occlude vessels causing thromboembolic diseases, such as stroke and myocardial infarction [1]. The coagulation system depends on sequential proteolytic activation of plasma-borne coagulation factors. Hepatocytes express and secrete most coagulation factors; therefore, bleeding and thrombotic diseases are common risks for liver disease patients. Factor XII (FXII) is a plasma protease that initiates the contact system, that in turn drives the proinflammatory kallikrein–kinin system and the so-called intrinsic pathway of coagulation. In addition to initiating thrombus formation and inflammation, FXII also triggers growth factor-like cell signalling [2]. Together, FXII contributes to vascular permeability, immune cell function, and proliferation with implications for liver inflammation and regeneration, and carcinogenesis. Here, we provide an overview of the FXII-driven contact system in liver inflammation and present current therapeutic approaches to prevent thrombo-inflammation.

## Factor XII-driven contact system

**Fig. 1:**
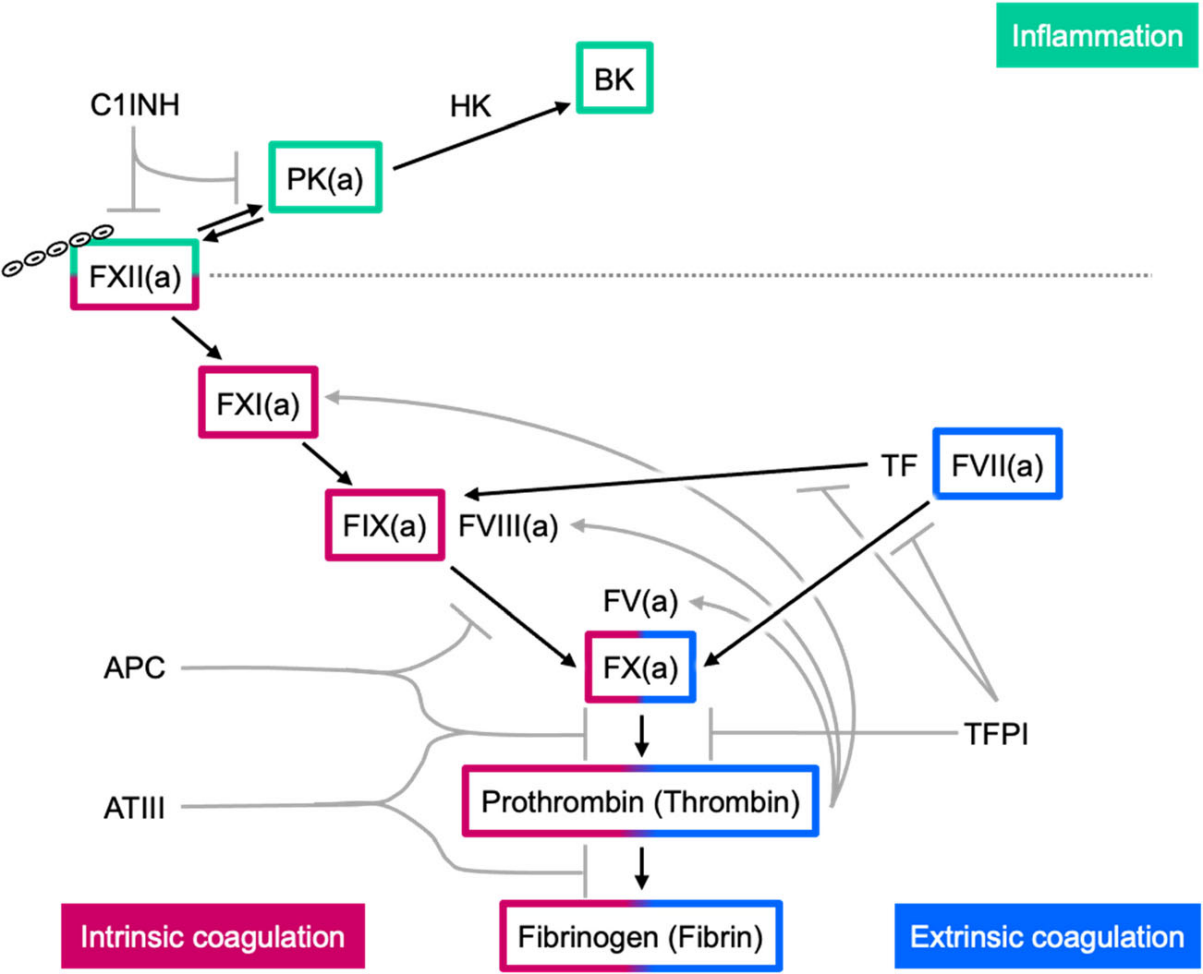
Overview of the plasmatic coagulation cascade. Negatively charged surfaces activate factor XII (FXII) to FXIIa, triggering proteolytic pathways of inflammation and coagulation. FXIIa cleaves plasma prekallikrein (PK) to plasma kallikrein (PKa), that in turn activates more FXII and liberates bradykinin (BK) from high molecular weight kininogen (HK). FXIIa and PKa are inhibited by C1 esterase inhibitor (C1INH). In addition, FXIIa activates factor XI (FXI) to FXIa that cleaves factor IX (FIX) to FIXa; FIXa then activates factor X (FX) in the intrinsic coagulation pathway. The extrinsic pathway starts with tissue factor (TF) that activates factor VII (FVII) to FVIIa, which leads then to FX activation. FXa generated by either pathway cleaves prothrombin to thrombin that eventually activates fibrinogen to fibrin and also FXI in a feed forward mechanism. In addition, factor VIII (FVIII) and factor V (FV) are cleaved by thrombin, thereby facilitating the activation of FX and prothrombin. The hemostatic balance is regulated by anticoagulant proteins that inhibit activated coagulation factors, such as activated protein C (APC) that blocks FVIIIa and FVa, antithrombin III (ATIII) that blocks primarily FXa and thrombin, and tissue factor pathway inhibitor (TFPI) that blocks the TF:FVIIa complex and FVa

Coagulopathy in liver disease is linked to unbalanced expression of plasma proteins that regulate FXII-driven intrinsic coagulation. The intrinsic pathway of coagulation is a proteolytic cascade of plasma serine proteases initiated by contact activation of zymogen FXII. FXII binds to negatively charged surfaces that induce conformational changes leading to autocatalytic cleavage and formation of an active serine protease, activated FXII (FXIIa) [3]. FXIIa-driven sequential activation of factor XI (FXI), factor IX (FIX), and factor X (FX) leads to the conversion of prothrombin to thrombin (assisted by a thrombin feed-forward activation of factor V (FV), factor VIII (FVIII) and FXI). Finally, thrombin mediates the cleavage of fibrinogen to fibrin, which aggregates to fibers and forms blot clots (Fig. 1). In contrast to intrinsic coagulation, mediated by FXIIa, the extrinsic pathway of coagulation is initiated through exposure of tissue factor (TF). TF binds and activates FVII to FVIIa, leading to a TF:FVIIa complex that activates FIX and FX and is inhibited by TF pathway inhibitor, which is primarily expressed by endothelial cells and platelets. Especially, Ca^2+^-dependent FVII, FIX, FX, and prothrombin require vitamin K-dependent carboxylation of glutamic acid residues by hepatic gamma-glutamyl-carboxylase. FV, FVIII, FXI, FXII, and FXIII, fibrinogen as well as the anticoagulant factors antithrombin III (ATIII) and proteins C and S are synthesized and released by the liver [4]. In contrast to all other coagulation factors listed above, FXII deficiency impairs thrombus formation in vivo [5], but is not associated with hemostatic abnormalities in mammals [6, 7]. Consistently, pharmacological inhibition of FXIIa provides thromboprotection without an increase in therapy-associated bleeding [8–10]. In addition to coagulation, FXIIa is a physiological activator of the kallikrein–kinin system that culminates in the generation of the proinflammatory mediator bradykinin (BK, a peptide hormone). FXIIa proteolytically cleaves plasma prekallikrein (PK), the precursor of the serine protease plasma kallikrein (PKa), and PKa then liberates BK from high molecular weight kininogen (HK). Furthermore, PKa and FXIIa engage in a feedback mechanism amplifying their proteolytic activation. BK binding to G protein-coupled B1 and B2 bradykinin receptors (B1R and B2R) mediates various proinflammatory effects such as vasodilation, pain sensation, and leukocyte adhesion and chemotaxis [2, 3, 11]. Impaired regulation of the kallikrein–kinin system leads to swelling disorders as seen in hereditary angioedema (HAE), a rare life-threatening disease with recurrent swelling episodes. HAE type I and type II are characterized by deficiency and dysfunctionality, respectively, of C1 esterase inhibitor (C1INH), the major inhibitor of both FXIIa and PKa. As a consequence, PK and HK levels decrease during acute swelling attacks, which instigated the development of B2R inhibitors to attenuate BK-mediated vascular permeability in HAE (see below). Notably, FXII mutations that increase susceptibility for autoactivation or prevent its inhibition through C1INH were identified as causative for HAE type III that has normal C1INH levels [12, 13].

## FXII signalling

**Fig. 2:**
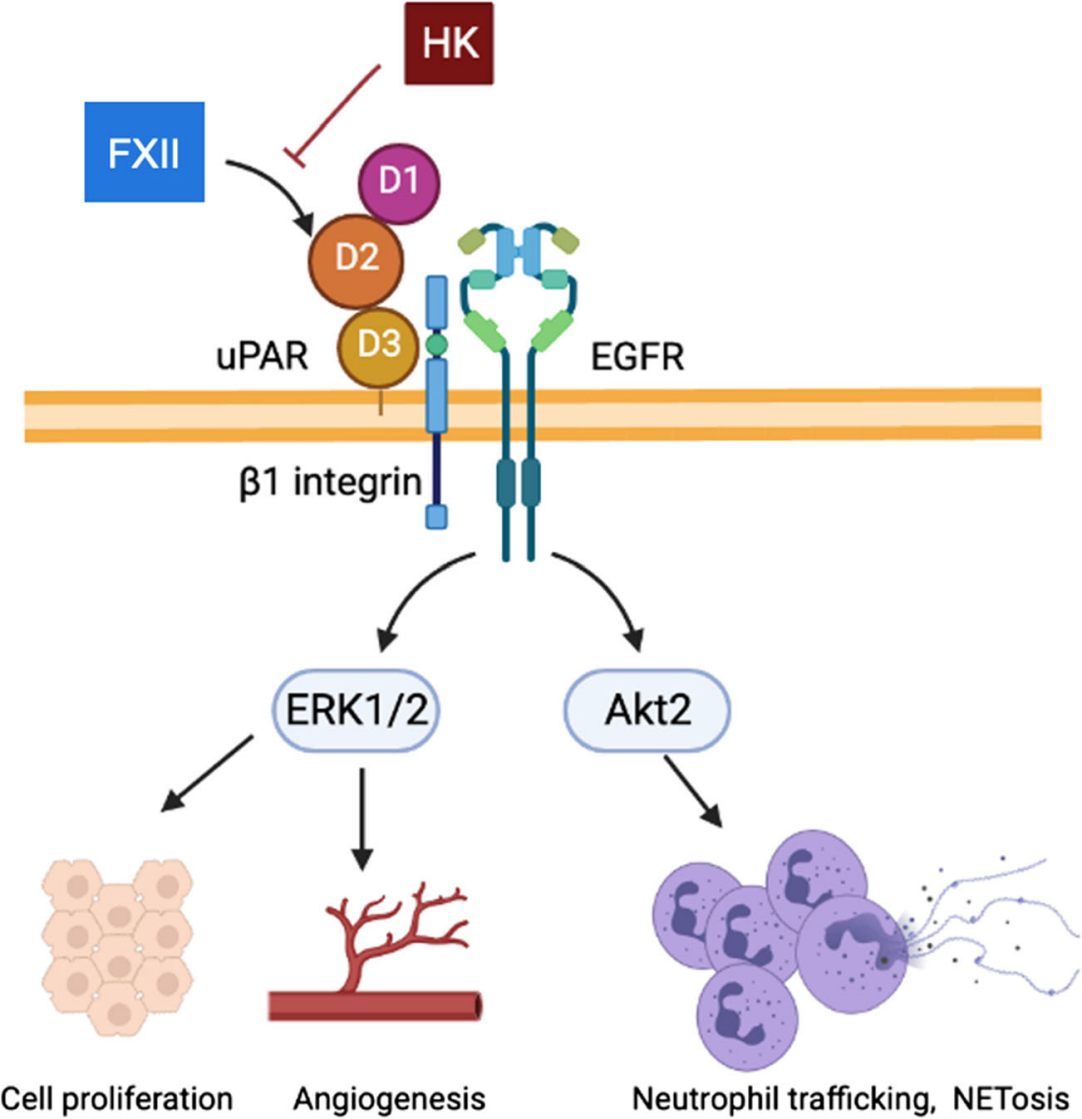
FXII signalling increases mitogenic activity, angiogenesis, and immune cell reactivity. Factor XII (FXII) binds to domain 2 (D2) of urokinase plasminogen activator receptor (uPAR). Signal transduction is facilitated by β1-integrin and epithelial growth factor (EGF), whereas high molecular weight kininogen (HK) competes with FXII for uPAR binding. FXII signalling results in (i) phosphorylation of extracellular signal-related kinase 1/2 (ERK1/2), leading to cell proliferation and angiogenesis and (ii) phosphorylation of Akt2, promoting neutrophil trafficking and neutrophil extracellular traps formation (NETosis). Modified from LaRusch et al. [17]

Intrahepatic inflammation and tissue repair are crucial processes for the clinical outcome after liver injury. Liver fibrosis and cirrhosis depend on immune cell recruitment and signal transduction pathways that promote hepatic fibrogenesis, such as urokinase plasminogen activator receptor (uPAR) signalling [14, 15]. In addition to FXIIa enzymatic activities, zymogen FXII has biological activity and mediates signalling through interaction of its two epidermal growth factor (EGF)-homologous domains in the N-terminal region with uPAR [16] (Fig. 2). FXII and HK compete for uPAR binding, whereby FXII mediates and HK prevents uPAR signalling [17]. FXII/uPAR interaction depends on Zn^2+^ ions that are released locally from both activated platelets and neutrophils [18]. Furthermore, β1-integrin and EGF receptor engage in FXII/uPAR signalling that mediates extracellular signal-related kinase 1/2 (ERK1/2) and AKT phosphorylation. Through these pathways, FXII zymogen exerts mitogenic activities in endothelial, alveolar, smooth muscle cells, and rat fetal hepatocytes [19, 20]. Due to its proliferative activity, FXII signalling promotes angiogenesis and it has been reported that FXII-deficient mice have fewer skin vessels [17]. Several studies found that FXII signalling induces proinflammatory cytokine production in alveolar cells [21], monocytes [22], macrophages [23], and dendritic cells [24]. The data suggest that FXII signalling plays a role in multiple inflammatory disease settings. Furthermore, recent findings showed that neutrophils express FXII, which promotes their activation in an autocrine manner. FXII deficiency in neutrophils interferes with migration and because the transfer of wild-type bone marrow restores this phenotype, it was shown that FXII from neutrophils, but not plasma, has a role in neutrophil-mediated wound healing [18]. FXII expression has also been found in lung fibroblasts in response to transforming growth factor-β (TGF-β) [25] and can be induced through steroid hormone binding to the *F12* promoter via estrogen-responsive elements [26]. Collectively, FXII signalling regulates cell activation in different tissues and promotes inflammatory responses.

The FXII/uPAR-mediated pathway in hepatocytes suggests that FXII autocrine signalling may participate in the complex regulation of hepatocyte proliferation and liver regeneration upon injury. Notably, liver disease has been associated with increased expression of TGF-β [27, 28], which was found to induce FXII in human lung fibroblasts [25]. At least for pulmonary fibrosis, FXII signalling has been shown to activate fibroblasts and might therefore also contribute to the fibrous obstruction and parenchymal loss in late-stage cirrhosis [29]. FXIIa drives proteolytic cleavage of pro-hepatocyte growth factor in vitro [30]. To what extent FXII signalling or FXIIa-mediated activation of hepatocyte growth factor contributes to liver regeneration in vivo remains to be addressed in future research as few studies have investigated the impact of contact system proteins on liver homeostasis so far. However, a recent study by Henderson et al. shows that deficiency in HK, but not FXII, FXI, or PK, protects from acetaminophen-induced liver injury [31]. HK cleavage exacerbates drug-mediated hepatotoxicity and promotes neutrophil recruitment and proinflammatory cytokine expression independently from B1R and B2R signalling. Thus, apart from FXII signalling, other contact system proteins may have a role in liver homeostasis.

## Contact activation

Proinflammatory and prothrombotic effects of the contact system promote endothelial dysfunction and hypercoagulability which has clinical relevance for liver disease patients. FXIIa initiates these pathways; thus, targeting contact activation that prevents the autocatalytic cleavage of FXII through contact with negatively charged surfaces appears as a promising therapeutic approach in liver disease. FXII and other contact system proteins have been found to assemble on membranes of endothelial cells [16], neutrophils [32], and platelets [33]. Activation of the latter cell types releases negatively charged mediators that trigger FXII contact activation and initiate the downstream proteolytic cascade. Fibrin formation triggered by FXII contact activation is the mechanistic basis of activated partial thromboplastin time (aPTT) clotting assays. For this, non-physiological polyanionic substrates (e.g., the white clay material kaolin, silica or ellagic acid) are used as FXII activators. Consistently, negatively charged biomolecules such as inorganic polyphosphate (polyP), polysaccharides, and DNA have been identified as surfaces for FXII contact activation in vivo [6, 34, 35]. The biopolymer polyP consists of linear chains of orthophosphates connected by energy-rich phosphoanhydride bonds. We recently found that polyP in platelets and megakaryocytes is regulated by the phosphate transporter xenotropic and polytropic retrovirus receptor 1 (XPR1) and that platelet-specific XPR1 deficiency causes polyP accumulation and hypercoagulability, and promotes venous and arterial thrombus formation [36]. Procoagulant polyP release has been shown for activated platelets, mast cells, and basophils [37].

In addition to polyP, exposure of another polyanion, extracellular DNA, has been identified to trigger FXII activation in vivo [38]. Several sources for extracellular DNA in circulation have been identified, including leukocytes, mast cells, disintegrating bacteria and viruses, and liver tumor cells [39–42]. Various pathogen-associated molecular patterns (PAMPs) and damage-associated molecular patterns (DAMPs) stimulate neutrophils to cast out their DNA into the extracellular space, forming neutrophil extracellular traps (NETs). Notably, FXII signalling contributes to the release of NETs, but is only facilitated after uPAR translocates to the plasma membrane upon neutrophil activation [18]. Similar to polyP, an array of studies showed that NETs have implications for thrombotic and inflammatory reactions in vivo [35, 43–45]. Soluble DNA, as well as NETs induced by glucose oxide or interleukin (IL)-8 stimulation of purified neutrophils, can bind and activate FXII in vitro [46]. Confocal microscopy of NETs induced by platelet-activated neutrophils showed that the DNA backbone of NETs associates with FXII [35, 46]. However, whether NETs directly trigger FXII contact activation or merely act as a scaffold for the assembly of FXII activators is still unknown [47]. Thrombin generation in the presence of NETs is reduced in FXII- and FXI-deficient plasma, indicating that the FXII–FXI axis mediates the procoagulant activity of NETs, at least in vitro [48]. In addition, we showed that NETs alone are sufficient for vascular occlusions during chronic inflammation in the absence of host enzymes DNase1 and DNase1L3 in vivo [49]. Underlining the importance of NETs for liver inflammation, DNase treatment or inhibition of NETs via genetic ablation of peptidyl arginine deaminase type IV reduces monocyte infiltration and cytokine production in an experimental steatohepatitis model [50]. Thus, cumulative evidence suggests that both platelet polyP release and NET formation provide a surface for FXII contact activation and are involved in pathophysiological liver processes [51, 52].

## Dysregulated coagulation in liver disease

The liver synthesizes and secretes most of the pro- and anticoagulant factors into the plasma. Both the extrinsic and intrinsic pathways rely on expression, post-translational modification, and the release of coagulation factors from hepatocytes. Accordingly, liver disease is associated with variable degrees of coagulation disorders due to alterations in the quality and quantity of coagulation factors [53]. The imbalance in mechanisms regulating coagulation is further enhanced by insufficient hepatic secretion of thrombopoietin, a hormone that induces megakaryocytes to form mature platelets [54]. Aside from decreased pro- and anticoagulant factors, stasis of venous blood flow through damaged liver tissue increases the risk for portal vein thrombosis (PVT) [4]. Thus, alterations of the hemostatic balance are multifactorial and an array of coagulopathies is associated with liver disease. Clinical diagnosis of the hemostatic state in liver patients is difficult because abnormal clotting test results often suggest a hemorrhagic coagulopathy when patients appear rebalanced due to the concomitant deficiency of both pro- and anticoagulant factors. Addressing the shortcoming of the prothrombin time (PT)-based international normalized ratio (INR) in liver patients, thrombin generation tests have been introduced that correct for anticoagulant protein C activity by addition of soluble thrombomodulin [55]. As a result, the perception of liver disease as an isolated risk for bleeding changed to a more complex view of liver disease-related coagulopathies including hemorrhagic and thrombotic components [56].

### Liver disease is associated with a procoagulant state

The severity of liver disease relates to the deficiency of coagulation factors and delayed clotting times. A prolonged PT is indicative for liver disease, and depletion of factors with a short half-life, e.g., FV and FVII, is used as a prognostic marker for acute liver failure (ALF) [53]. In contrast to ALF, increased PT in chronic liver disease is linked to reduced FVII expression and commonly assessed in prognostic indices, such as Child-Pugh and Mayo end-stage liver disease [57]. Further disease progression additionally prolongs aPTT by insufficient liver synthesis of FXII, FXI, PK, and HK [58]. In mild and moderate cirrhosis, fibrinogen may increase as an acute phase reactant in plasma [59], whereas patients with end-stage liver diseases may display decreased or functionally abnormal fibrinogen [60]. Contrary to clotting test results, bleeding disorders in liver disease patients are uncommon (except for spontaneous leakage and rupture of varices), since synthesis of both pro- and anticoagulant factors is decreased in cirrhosis. However, the significance of thrombotic diseases in cirrhotic patients [61, 62] and a hypercoagulable state in patients with primary biliary cirrhosis and primary sclerosing cholangitis have been reported [63, 64]. Moreover, local venous thrombosis has been attributed to increased liver inflammation and fibrosis, thereby promoting disease progression [65].

### Chronic liver inflammation promotes coagulation disorders

Heterogenic etiologies of chronic liver inflammation entail distinct alterations of the contact activation pathway. Non-alcoholic fatty liver disease (NAFLD) is the most common liver disease in the general population. One study showed that 46% of NAFLD patients have thrombotic risk factors that correlate with the extent of hepatic fibrosis [66]. Circulating FVIII, FIX, FXI, and FXII activities were found to be increased in subjects with NAFLD compared to those without NAFLD [67], underlining the impact of the coagulation system for liver disease pathogenesis. Moreover, NAFLD is accompanied by excessive synthesis of cholesterol and free fatty acids, leading to increased release of very-low-density lipoprotein (VLDL) particles into the circulation. The lipoprotein component phosphatidylethanolamine (PE) has been found to trigger FXII contact activation in thrombin generation assays [68], suggesting that elevated phospholipid levels contribute to blood coagulation in dyslipidemia.

In contrast to NAFLD, a direct link between viral hepatitis and impaired coagulation is less clear [69]. However, as with other liver diseases, hepatitis is associated with cardiovascular disease through dysregulated lipoprotein metabolism, which may facilitate PE-driven FXII contact activation and thrombo-inflammation [70, 71]. Consistent with aggravated immune responses, hepatitis is also associated with the occurrence of antiphospholipid syndrome, an autoimmune disorder that increases thrombosis risk through autoantibodies targeting coagulation factors [72]. Besides antiphospholipid syndrome, systemic lupus erythematosus is often diagnosed in patients with autoantibodies that interfere with clinical coagulation tests. Furthermore, case reports in which chronic liver disease patients develop specifically anti-FXII autoantibodies indicate that inhibition of the intrinsic coagulation can also occur secondary to liver inflammation [73]. Thus, inflammatory and thrombotic mechanisms influence each other mutually in chronic liver diseases.

In addition to the procoagulant balance, patients with advanced liver disease show a prevalence for hyperfibrinolysis with low levels of α2-antiplasmin inhibitor and decreased hepatic clearance of tissue plasminogen activator [53]; however, its clinical relevance has been disputed and it is not clear whether changes are directly induced by liver disease or secondary to clotting activation [56]. Notably, consumption of plasminogen increases in ALF and the fibrinolytic protease plasmin exerts detrimental effects on acetaminophen-induced ALF through HK cleavage [31, 74], suggesting that thrombo-inflammatory pathologies in ALF are associated with extrinsic pathway activation.

### Platelet and neutrophil activation in liver disease

In the light of reduced contact system protein synthesis and procoagulant and proinflammatory imbalance in liver patients, the presence of polyanions that drive contact activation has implications for disease progression and hemostatic state. Chronic liver disease is associated with mild to moderate thrombocytopenia and platelet counts fall rarely below the range of 30,000–40,000/mm^3^ [53]. Altered platelet activatability in liver disease has remained controversial, whereas the formation of platelet–leukocyte complexes is consistently found to be increased [75]. It is currently unknown to what extent platelet-derived polyP increases contact activation in liver disease; however, a comparative study provided evidence for increased platelet activation in cirrhosis through low-grade endotoxemia [76]. On the other hand, formation of neutrophil extracellular traps (NETosis) is increased in acute and chronic liver disease models and aggravates liver injury [77], suggesting that increased activation (e.g., via platelet-neutrophil complexes) and impaired NET clearance contribute to procoagulant and proinflammatory functions of the contact system in liver disease. Notably, FXII signalling in neutrophils stimulates cell adhesion, migration, and NETosis [18], indicating that FXII facilitates its activation via the induction of NETosis that may contribute to a FXIIa-driven procoagulant state in liver disease.

### Malignancies and extracellular vesicles promote thrombotic diseases

Chronic liver damage has been associated with an increased risk for liver cancer. While some of the mechanisms that affect the contact system in liver disease may persist in liver cancer patients, others emerge that additionally play a role for the hemostatic state in liver cancer patients. Hepatocellular carcinoma (HCC) is the most common primary liver cancer with an 85–95% prevalence for liver cirrhosis among HCC patients [78]. HCC is the fourth leading cause of cancer-related deaths as it is usually diagnosed at a late stage, thereby limiting therapeutic options [79]. HCC patients have an increased risk for PVT and like other cancers, HCC is also associated with a higher incidence of systemic venous thromboembolism (VTE) [80, 81]. Similar to patients with advanced liver disease, synthesis of coagulation factors and their cognate inhibitors are reduced in HCC patients leading to coagulation disorders that may even change during disease progression. Moreover, endothelial activation and hemodynamic alterations by HCC growth can tip the hemostatic balance towards hypercoagulability. Furthermore, cancer cells can directly promote the coagulation cascade through the production of procoagulant factors, and proinflammatory and proangiogenic cytokines, and by interaction with endothelial and blood cells [82]. Early studies indicated that cancer cells have an impact on contact system activation. FXII, PK, and HK decrease in plasma of patients with metastatic liver cancer and increased contact system activation has been found in ascites from cancer patients [83, 84].

A relatively small number of studies investigated the contact system in liver cancer. However, several lines of evidence suggest that HCC exerts FXII signalling and influences contact activation. Firstly, in HCC-derived HepG2 cells, but not enteroendocrine L cells, FXII and FXIIa promote proliferation [20], suggesting a specific effect of FXII/FXIIa signalling for liver cell proliferation. Further research will show whether FXII/FXIIa regulates HCC growth and liver fibrosis in vivo. Secondly, consumptive deficiency of contact system proteins in ascites and plasma from cancer patients suggests that the tumor microenvironment modulates FXII expression, activity, and activation [83, 84]. Notably, cancer cell-associated urokinase-type plasminogen activator (uPA) that drives the generation of plasmin activates FXII and PK in vitro and uPA expression has been shown to determine HCC tumor recurrence [85, 86]. The underlying mechanism remains to be shown but these results indicate a possible role for FXII in uPA-driven growth of HCC. Expression of FXII by HepG2 cells is inhibited through the proinflammatory cytokine IL-6, and it has been suggested that FXII acts as a negative acute phase protein [87]. Indeed, the reduction of FXII and PK plasma levels has been reported in a murine sepsis model, in which depletion of PK, but not FXII, is associated with bacterial growth inhibition in *Streptococcus pyogenes* sepsis [88]. Thus, limiting contact system proteins in response to inflammation may have a beneficial effect on anti-HCC immune responses as IL-6 inhibits FXII expression and potentially decreases FXII signalling.

In addition to soluble factors, cancer cells contribute to contact activation and cancer-associated thrombosis through extracellular vesicles (commonly referred to as microvesicles shed by cancer cells as well as endothelial cells, platelets and leukocytes). Microvesicles are heterogeneous, nano-sized vesicles that carry a variety of bioactive molecules (e.g., proteins, mRNA, miRNA, DNA, and lipids) and expose procoagulant factors on their surface [89, 90]. Microvesicles promote coagulation through several mechanisms. Firstly, they expose phosphatidylserine (PS) that supports the assembly of coagulation factors. Likewise, FXII binding, and the consecutive start of the intrinsic coagulation cascade through PS externalization, is mediated by apoptotic cells that are more frequent in proliferating tumors [91]. Secondly, microvesicles express TF on the surface that drives the extrinsic pathway of coagulation and thirdly expose polyP that initiates the contact system [78, 92]. The latter has been shown for prostasomes (microvesicles secreted by prostate cancer cells) that are rich in polyP with a chain length of 200–1000 phosphate moieties. Prostasomes increase thrombin formation in a FXII-dependent manner in prostate cancer patients, whereas genetic and pharmacological inhibition of FXII and polyP abrogates prostasome-induced thrombus formation in vivo [93]. Thus, cancer-associated microvesicles induce a hypercoagulable state through the extrinsic and intrinsic coagulation systems. In cirrhotic HCC patients, the level of endothelial, platelet, leukocyte, and PS-exposing microvesicles is increased compared to that in cirrhotic patients [94], suggesting that HCC aggravates hypercoagulability.

**Fig. 3:**
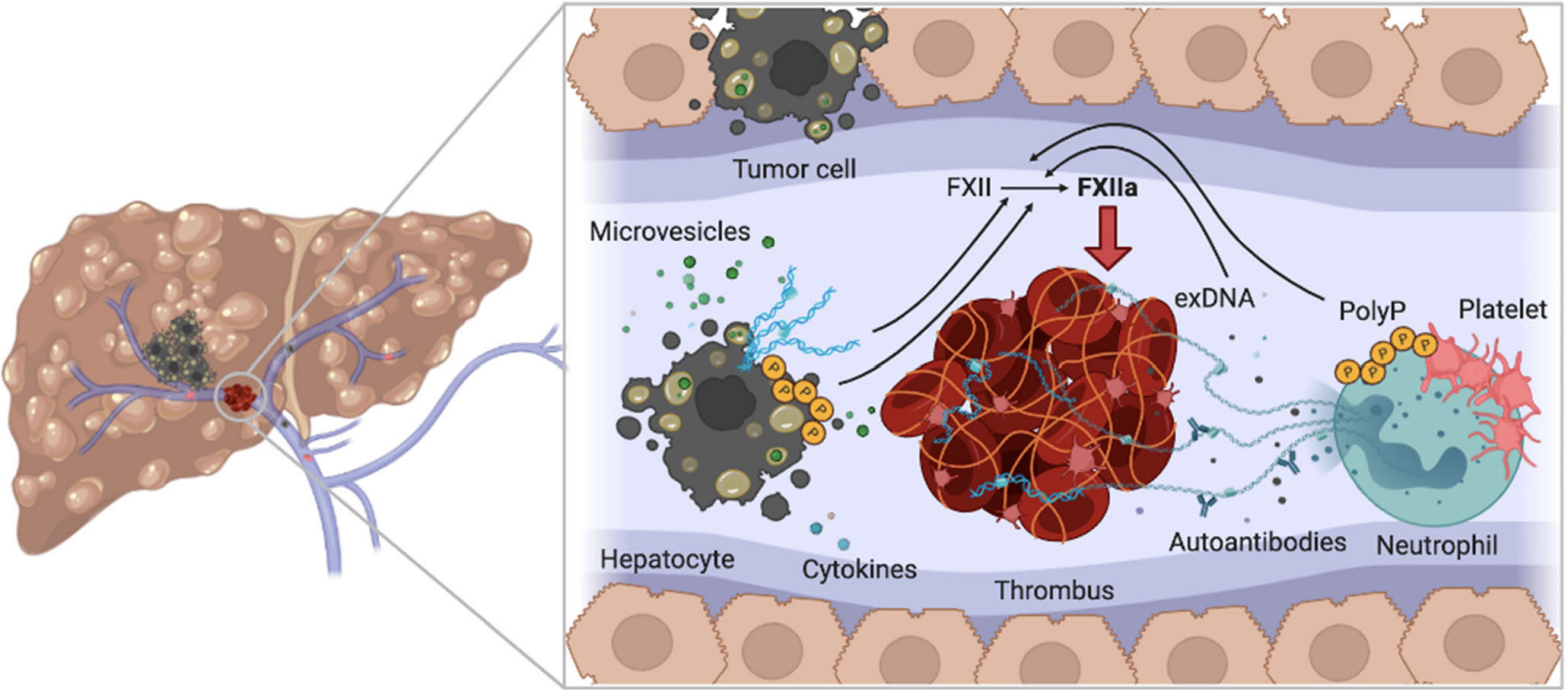
Contact activation in liver disease. Liver disease is associated with a hypercoagulable state, with portal vein thrombosis being the most common thrombotic comorbidity. Polyphosphate (polyP) and extracellular DNA (exDNA) released from hepatocellular carcinoma cells and tumor-derived microvesicles trigger contact activation of factor XII (FXII). Proinflammatory cytokines recruit immune cells, such as neutrophils that increase exDNA through neutrophil extracellular trap formation. Chronic liver inflammation leads to cirrhosis, blood flow alteration, apoptosis, and autoantibodies that contribute to the procoagulant condition and injury of the hepatic vasculature. Platelet activation causes polyP release that triggers FXII activation, leading in turn to thrombus formation via the intrinsic pathway of coagulation

Besides microvesicles, other mediators for contact system activation are abundant in cancer. Tissue necrosis and tumor apoptosis elevate the level of polyP and extracellular DNA in the circulation, originating from the tumor, non-tumoral cells (e.g., neutrophils), and chemotherapy-associated apoptotic cells [95]. Cancer cell-derived extracellular DNA and cytokines induce neutrophilia and activate NET formation, thereby contributing to tumor-associated thrombotic diseases [96]. Moreover, in a recent study by van der Windt et al., NETs have been shown to promote the development of HCC in liver disease [50]. Thus, NETosis and cancer progression influence each other with implications for the cancer-associated hypercoagulable state (Fig. 3).

## Therapy and anticoagulation in liver diseases

Systemic inflammation, advanced age, immobility, and low levels of endogenous anticoagulants make patients with liver disease, especially decompensated cirrhosis, more prone to VTE, PVT, atrial fibrillation, and other thrombotic complications [56]. Novel treatment strategies for liver disease-associated coagulation disorders are needed. Inhibition of FXII provides a safe target for thromboprotection without increasing bleeding events [11]. Neutralization of FXIIa and interference with polyP-dependent FXII activation prevent pulmonary thromboembolism, ferric chloride-induced arterial thrombosis, and prostate cancer-associated venous thrombosis in animal models [10, 36, 93]. Moreover, clinical trials are currently performed with FXIIa-blocking antibodies CSL312/garadacimab (CSL Behring). Targeting FXIIa might be beneficial for diseases with aberrant FXIIa activity, e.g., the kallikrein–kinin system in HAE patients and thrombosis-related complications in SARS-CoV-2 infections [97]. Especially, the absence of bleeding tendency by FXIIa blockade may support its use as a safe antithrombotic treatment in liver disease because conventional direct oral anticoagulants that prevent frequently occurring PVT in early-stage disease will become inapplicable in patients with late-stage chronic liver disease [98].

Furthermore, regulating BK signalling in liver disease potentially interferes with inflammation, angiogenesis, proliferation, and tumor autocrine signalling [99]. Consistently, the B2R antagonist icatibant (Firazyr, Takeda) has been approved for HAE and is in trials for several other indications, such as heart failure and SARS-CoV-2 infection, while various other B1R and B2R antagonists are also currently investigated in cancer [99]. Notably, although anti-inflammatory drugs may mitigate procoagulant processes, their use in liver disease patients has not been recommended because of metabolic complications (e.g., hyperlipidemia and hypertriglyceridemia induced by inhibitors of calcineurin and mammalian target of rapamycin) [100]. The degradation of NETs offers another option to reduce inflammation and coagulation via the FXII/NET-axis. Exogenous DNase is used to degrade the DNA backbone of NETs in cystic fibrosis [101] and has been shown to prevent NET clots [49], the progression from liver disease to HCC [50], and cancer cell activation in mice [102].

Overall, liver diseases still pose a large unmet medical need. Novel treatments that reduce hepatic inflammation and reverse coagulation disorders while retaining metabolic liver function in patients are required. Targeting the contact system has the potential to mitigate thrombo-inflammation. In particular, inhibition of FXIIa and NETosis may help early-stage liver disease patients, as this could ameliorate hypercoagulability and decelerate cirrhosis progression, without interfering with hemostasis.

## Conclusion

The FXII-driven contact system promotes inflammation and cell signalling; its direct contribution to liver disease however remains to be elucidated. FXII signalling induces mitogenic activity and angiogenesis, whereas the proteolytic activity of FXIIa triggers BK-mediated inflammation and elicits a prothrombotic state. In liver disease and HCC, coagulation factor synthesis is impaired and a dysregulated contact system potentially contributes to disease progression. Targeting mediators of contact activation like polyP and extracellular DNA poses a novel opportunity for thrombosis prevention including hypercoagulability in liver diseases. Further research is needed to elucidate thrombo-inflammatory mechanisms and to stratify liver disease patients in relation to their thrombotic risk.
